# Nutritional Status and the Outcomes of Endoscopic Stenting in Benign and Malignant Diseases of Esophagus

**DOI:** 10.3390/nu15061524

**Published:** 2023-03-21

**Authors:** Wojciech Dudzic, Cezary Płatkowski, Marcin Folwarski, Jarosław Meyer-Szary, Karolina Kaźmierczak-Siedlecka, Marcin Ekman, Tomasz Wojciechowicz, Marek Dobosz

**Affiliations:** 1 Department of General and Gastrointestinal Surgery and Nutrition, Copernicus Hospital Gdansk, 80-336 Gdansk, Poland; 2 Department of Clinical Nutrition, Medical University of Gdansk, 80-210 Gdansk, Poland; 3 Department of Paediatric Cardiology and Congenital Heart Defects, Medical University of Gdansk, 80-210 Gdansk, Poland; 4 Department of Medical Laboratory Diagnostics—Fahrenheit Biobank BBMRI.pl, Medical University of Gdansk, 80-210 Gdansk, Poland; 5 Department of Surgical Oncology, Medical University of Gdansk, 80-210 Gdansk, Poland

**Keywords:** esophageal constriction, endoscopically stenting, esophageal cancer, palliative stenting, dysphagia, malignant constriction of the esophagus

## Abstract

Background. Endoscopic stenting (ES) is a widely known method for palliative dysphagia treatment in esophageal strictures. Esophageal cancer is often associated with advanced malnutrition, which may increase the risk of complications of the procedure. The aim of this study was to evaluate complication rates and the impact of nutritional status on the outcomes of ES. Patients and Methods. A single-center retrospective study was conducted at Copernicus Hospital in Gdańsk, Poland. Adult patients who underwent endoscopic stenting between February 2014 and December 2018 were included. The influence of patient characteristics (age, sex, indications for esophageal stenting, and location of stenosis) and nutritional status (BMI, NRS 2002, GLIM, and dysphagia score) on complication rates and survival were analyzed. Results. Eighty-one patients (69% men) were enrolled in the study. In 69%, the indication for ES was malignancy (mainly esophageal cancer). The median dysphagia score significantly decreased from 2.8 to 0.6 after the procedure (*p* < 0.001). Complications were observed in 27% (*n* = 22) of the patients. Early complications were bleeding (2.5%), stent unexpansion (2.5%), and stent migration during the procedure (3.7%). There were no early fatal complications of the procedure. Late complications included: stent migration (6.2%), tissue overgrowth (6.2%), food impaction (2.2%), fistula formation (3.7%), bleeding (3.7%), and stent malposition (1.2%). A total of 76% of the participants scored ≥ 3 points in nutritional screening (NRS2002) and 70% were diagnosed with severe malnutrition (GLIM -stage 2). A stent diameter of < 2.2 cm compared with ≥ 2.2 was associated with a higher rate of migrations (15.5% vs. 2.5%). The median survival time in the malignant group was 90 days. Histopathological diagnosis and patients’ nutritional status (BMI, NRS 2002, GLIM, and dysphagia score) had no significant effect on complication rates and survival after esophageal stent insertion. Conclusions. Endoscopic stenting is a relatively safe procedure for the palliative treatment of esophageal strictures. Severe malnutrition, although common, does not affect the outcomes of the procedure.

## 1. Introduction

The first attempts to treat esophageal strictures by stents date back to the 19th century, when Symonds introduced the first stent made of ivory and silver [1]. Since then, numerous advances have been introduced in the technique of endoscopic stenting (ES) and in stent materials. This has led to a variety of new indications, most notably malignant, postoperative or post-inflammatory strictures, congenital esophageal atresia, and chemical burns. Other endoscopic techniques such as ballooning, mechanical dilatation, or intralesional injection of steroids are currently the methods of choice for benign diseases [2,3], with ES recommended after repeated failures [4]. Other indications for ES include esophageal fistulas or esophageal wall perforations [5,6] and acute variceal hemorrhage [7]. The incidence of esophageal strictures was estimated at 1.1 per 10 000 person-years in the United Kingdom between 1994 and 2000, and increased with age. The majority of cases were peptic and malignant strictures [8].

Malignant dysphagia is a common problem in patients with esophageal cancer, the seventh most common cancer and the sixth cause of death worldwide [9]. Its natural progression leads to narrowing of the esophageal lumen. Gastric, lung, breast, and mediastinal cancers, both primary and metastatic, can also cause dysphagia and external compression of the esophageal lumen. The gradual development of swallowing problems, initially with solid food and eventually even with saliva, negatively affects the quality of life and nutritional status [9]. In the cross-sectional study by Van et al. (in 206 newly diagnosed patients with esophageal cancer), 87.4% of the patients were found to suffer from dysphagia. A total of 52.9% of the patients suffered from mild/moderate malnutrition, and 29.6% from severe malnutrition. Patients with dysphagia were 3.39 times more likely than patients without dysphagia to lose >10% of their body weight in one month [10]. In the Cao et al. study regarding 1482 patients, it was noted that malnutrition occurred in 76% of the patients with esophageal cancer (using the PG-SGA screening method—Patient-Generated Subjective Global Assessment—score ≥4) and in 50% of the patients (using the NRS 2002 method—Nutritional Risk Screening—score ≥3) [11]. The European Society for Clinical Nutrition and Metabolism (ESPEN) indicates that patients with upper GI cancer are at risk for malnutrition and recommends a protein intake of more than 1 g/kg/day, and up to 1.5 g/kg/day, if possible, and an energy provision of 25–30 kcal/kg/day [12]. A study of patients with esophageal cancer, patients during chemoradiotherapy (CRT) confirmed inadequate energy intake before, immediately after, and in a 4–6-week follow-up in 55.7%, 58.7%, and 27.3%, and protein intake inadequacy in 89.8%, 89.1%, and 72.7% of cases, respectively. Mean energy and protein intake after CRT were 18.3 ± 11 kcal/kg/day and 0.66 ± 0.5 g/kg/day [13]. Failure to provide effective oral nutrition can lead to loss of muscle mass and function, which was confirmed in the study from Keio University Hospital (Tokyo, Japan). A total of 71% of the patients with esophageal cancer met the criteria for sarcopenia based on the measurement of the cross-sectional area of the psoas on the computed tomography before the treatment. Patients with esophageal cancer who had sarcopenia preoperatively were more likely to develop, often, dysphagia in the postoperative period compared with non-sarcopenic patients [14].

ES may be considered as a palliative or bridging therapy for patients with a planned brachytherapy [4,15,16,17,18]. In the case of a potentially curative surgery or radio/chemotherapy, ES may adversely affect the outcomes of planned oncologic treatment [4,19,20]. Esophageal cancer is associated with a high prevalence of malnutrition, which affects the treatment outcomes [21,22,23,24]. To our knowledge, there are no studies focusing on the effect of poor nutritional status on complication rates of ES.

There is a wide range of indications and potential clinical applications for esophageal stenting in significant population of patients. Therefore, specialized endoscopic units providing the treatment should be widely available. The aim of this retrospective study was to evaluate both the clinical and technical success and the safety of ES in patients with benign and malignant diseases of the esophagus in a single center of a district hospital.

## 2. Materials and Methods

In a single-center retrospective study, we enrolled adult patients who underwent endoscopic esophageal stenting between February 2014 and December 2018 at the Department of General Surgery, Nicolaus Copernicus Hospital in Gdansk. The study was approved by our Institutional Review Board (NR: KB-20/22). Data on nutritional status, indication for stent implantation, histopathological diagnosis, stent size, complications, and reinterventions were collected from medical records. The Polish Ministry of Digital Affairs provided patient survival data in December 2021. Localization of proximal onset of stenosis was determined by dividing the esophagus into three sections: upper (i.e., 20–28 cm), middle (29–36 cm), and lower (≥37 cm), measured from the incisors.

The influence of stent characteristics, nutritional status, and the principal diagnosis on procedural complication rates and overall survival (in malignant patients) was evaluated. Complications were divided into early (perioperative and during hospitalization) and late (tissue overgrowth, migration, bleeding, food impaction, fistula formation). The influence of stenting on the dysphagia relief was analyzed. Age, stent length, diameter, and BMI were subdivided in relation to the median cut-off values.

### 2.1. Assessment of Nutritional Status

Prior to the procedure, the following data were collected: body mass index (BMI) and Nutritional Risk Score (NRS 2002). The severity of malnutrition was determined according to GLIM criteria (Global Leadership Initiative on Malnutrition). According to the NRS 2002, we assessed impaired nutritional risk (0–3 points) and severity of disease—increase in energy requirements (0–3 points). Patients older than 70 years received an additional point.

GLIM (Global Leadership Initiative on Malnutrition) [25]. To diagnose malnutrition, at least one etiological criterion (reduced food intake, inflammation) and one phenotypic criterion (involuntary weight loss, reduced muscle mass, body mass index) had to be met. Based on these parameters, patients were assigned to moderate malnutrition (S1) or severe malnutrition (S2).

Dysphagia was assessed according to the five-grade scale, i.e.:

- 0—normal swallowing;
- 1—some solid;
- 2—semi-liquid;
- 3—fluids;
- 4—complete dysphagia.

BMI (body mass index) was divided into four categories:

- <16—severe thinness;
- 16–18.49—underweight mild-moderate thinness;
- 18.5–24.99—normal range;
- 25–29.99—overweight;
- ≥30—obesity.

### 2.2. Stent Placement Technique

A standard endoscopic procedure was used to place the stent. A radiological method was used to measure the stricture. The size of the stent was estimated to cover the entire stricture with a margin of 2 cm on both sides of the stent (except for high-grade strictures). In narrow stenoses, balloon dilatation was performed before the procedure. The procedure was considered successful if the stent was expanded in the correct location. The following stent brands were used: Teawoong Medical (Gimpo-si, South Korea), Endo-flex (Voerde, Germany), Microtech (Fletcher, NC, USA), and Changzhou Health Microport Device System (Changzhou, China). The stent lengths varied from 60 to 140 mm and the diameter from 18 to 24 mm.

### 2.3. Statistical Analysis

Statistical analyses were performed using Wizard Pro 2.0.12 (Evan Miller, Chicago, IL, USA). Normality of the distribution was tested using the Shapiro–Wilk test. Data are presented as mean (±SD) for normally distributed data or median (min—max). Prevalence data are expressed as percentages with precision of two significant figures. The Student’s *t*-test and Mann–Whitney test were used, as indicated. For multiple comparisons, proper ANOVA or Friedman tests were used. Categorical data were compared with the chi-square test and the chi-square test for trends (Cochran–Armitage). Pearson’s correlation, linear regression, and multivariable linear models were used. Survival function was estimated using the Kaplan–Meier estimator and survival data between the groups was compared using the logrank test. Results with a *p*-value < 0.05 were considered statistically significant and are marked with asterisk (*).

## 3. Results

A total of 81 patients were included in the analysis. All procedures were considered successful, with a mean stenting time of 28.9 min. A total of 69% of the patients were men. Before stenting, the majority of the patients (61%) suffered from grade 3 or higher dysphagia. Seventy-six percent of the participants scored at least 3 points in NRS 2002 and 70% of the patients were diagnosed with severe malnutrition (grade 2 according to the GLIM criteria). The average weight loss in the last 6 months was 13.2 kg corresponding to 23.2% of body weight (2–60%). Malignant disease was the cause of stricture in 69% of the patients, most commonly (75%) esophageal cancer (squamous cell carcinoma—SCC in 68.7%). The most common site for stent implantation was the upper esophagus (38%) with a mean stenosis length of 5.2 cm (SD ± 2.04 cm). Among patients with a benign diagnosis, 36% underwent stenting for perforation of the esophageal wall, 32% for post-inflammatory stenosis, and 24% for chemical burn. The baseline characteristics of the patients are shown in Table 1.

**Figure 1:**
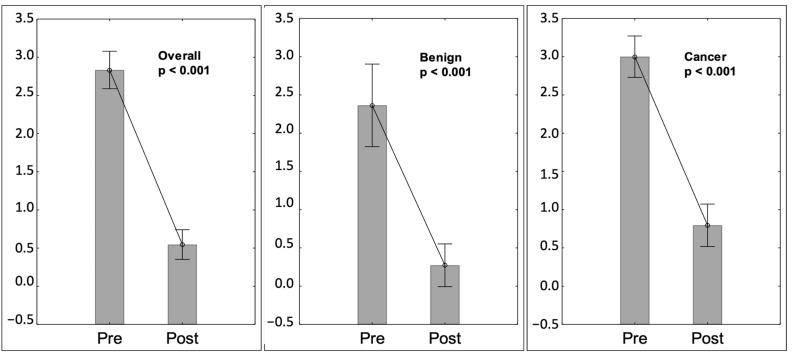
Dysphagia. Pre—prior to procedure, Post—two days after the procedure.

The mean dysphagia score two days after the procedure was 0.6 (±0.84) and was significantly lower (*p* < 0.001) than before the procedure—mean 2.8 (±1.08). Median dysphagia relief was 3 (±1.23) (Figure 1).

Complications were observed in 27% (*n* = 22) of the patients (malignant diagnosis was 22%, *n* = 18; benign 4.9%, *n* = 4). Early complications were bleeding (overall: 2.5%, *n* = 2; malignant 1.2%, *n* = 1; benign 1.2%, *n* = 1), stent unexpansion (2.5%, *n* = 2; all malignant), stent migration during the procedure (3.7%, *n* = 3; all malignant). There were no early fatal complications of the procedure. Late complications: stent migration (overall: 6.2%, *n* = 5; malignant 2.5%, *n* = 2; benign 3.7%, *n* = 3), tissue overgrowth (6.2%, *n* = 5; all malignant), food impaction (2.5%, *n* = 2; all malignant), fistula formation (3.7%, *n* = 3; all malignant), bleeding (3.7%, *n* = 3; all malignant), and stent malposition (1.23%, *n* = 1; all malignant). The median time from the procedure to restenosis due to tissue overgrowth was 168 days (61–173). Esophageal bleeding was observed in 3 patients (3.7%) after a median time of 27 days (24–62) and resulted in patient death. Migration occurred after a median of 56 days (41–121). The median time from stent implantation to removal in the benign patients was 76.1 days. All complications were treated endoscopically, and no patients required surgical intervention.

There was no significant effects of age, sex, histopathologic diagnosis, and nutritional status (BMI, NRS 2002 score, GLIM, and degree of dysphagia) on complication rates after esophageal stent placement. A stent diameter of < 2.2 cm compared with ≥2.2 was associated with a higher rate of migrations (15.5% vs. 2.5%) (Table 2 and Table 3).

## 4. Discussion

ES in experienced centers is an effective method for treating swallowing disorders caused by strictures. In the present study, all procedures were rated as a technical success with a median dysphagia relief of 3 (±1.23). Similar results are confirmed in other studies. In a systematic review (66 studies, *n* = 1752), Kamarajah et al. showed that the clinical success rate of esophageal stents was 87%, while the technical success rate was 96% [26]. Chandan et al. showed a technical success rate of 94.7% and a pooled clinical success rate of 82% for palliative ES without fluoroscopy [27]. A total of 24% of benign strictures in our study were caused by chemical burns as a result of accidental or intentional ingestion of a corrosive substance. This remains a major problem in developing countiries. Caustic ingestion leads to esophageal mucosal damage and strictures in up to 22% of cases depending on the type and amount of the substance digested [28]. The World Health Organization estimates 110 cases per 100,000 citizens are treated for this reason [29]. In France, 15,000 cases are noted annually with a mortality of 10% [30]. Panigrahi et al. investigated the most common causes of dysphagia. A total of 216 consecutive patients admitted to the gastroenterology outpatient department with a history of dysphagia were included in the study. Benign strictures were diagnosed in 31.48% (corrosive injury in 70.59%, peptic stricture in 11.76%, postoperative strictures in 5.88%) and esophageal carcinomas in 23.62% [31].

With the development of endoscopic technique and stent material, the procedure of ES became safer and more accessible to patients [32,33,34]. Nevertheless, several complications must be considered. In a study by Schoppmann et al., it was found that for self-expanding long-term esophageal stents, the rate of overall complications was 30% and the rate of reinterventions was 17% [35]. Turkyilmaz et al. studied complications associated with metal stent placement for malignant esophageal strictures [36]. Among 174 patients, bleeding was observed in 11 patients, aspiration pneumonia in 6 patients, tracheal compression in 3, perforation in 2 and esophago-respiratory fistula in 2 patients. Reintervention was required in 32% of the patients [36]. In our study, the rate of late complications was 23% in malignant strictures and 7.1% in benign. The most common late complication in patients with malignant stricture was restenosis due to tissue overgrowth as a result of cancer development (8.9%). It has been reported in other studies in 2.5% to 36% of the patients [4,37,38].

ES requires retraction of the esophageal wall affected by disease, which carries the risk of perforation, observed in 2% to 8% of the patients [4,39,40]. In the present study, in 5 patients (8.9%) esophageal fistula was a primary indication for ES, and only 2 patients developed fistula as a result of stenting. Another life-threatening complication is bleeding, which can be observed in 2.0% to 21% of the patients after ES [4,40,41]. In the present study, 3 patients (5.4%) were readmitted to the hospital for severe bleeding from the esophagus, resulting in patient death. However, in these cases, advanced cancer ulcerations were observed and bleeding occurred more than 3 weeks after ES (24–64 days). Therefore, the direct association between ES and bleeding is not certain. Fuccio et al., in a systematic review and meta-analysis, evaluated the outcomes of stent implantation in refractory benign esophageal stricture (RBES) [42]. The efficacy of the procedure was reported to be 40% with stent migration in 29%. ES is an effective procedure in palliative dysphagia, supporting the possibility of oral feeding during neoadjuvant treatment [43] or brachytherapy [4,15,16,17,18]. Preoperative stenting during neoadjuvant therapy for esophageal cancer in patients planned for curative surgical treatment improved dysphagia scores after the procedure but had no beneficial effect on weight, body mass index (BMI), or albumin in the recent systematic review by Ahmed et al. ES was associated with a lower R0 resection rate and overall survival [44]. This was confirmed by a study from Finland, which showed worse five-year survival and ninety-day outcomes after preoperative ES [45]. In cancer patients undergoing chemotherapy, stent migration may occur more frequently due to the reduction in tumor mass in response to oncologic treatment. In a systematic review and meta-analysis, Nagaraja et al. evaluated both the safety and efficacy of esophageal stents prior to neoadjuvant treatment [43]. A total of 51% of the patients reported chest discomfort and 33% experienced stent migration. In our data, stent migration occurred in 7.1% of benign and 3.6% of cancer patients. In two cases (3.6%), the stents were obstructed by food. This problem required additional endoscopic treatment. Other studies have shown it may be observed in 2.0% to 27% of the patients [4,40,46]. A stent diameter of < 2.2 cm compared with ≥2.2 was associated with a higher rate of migrations (15.5% vs. 2.5%) in the present study. Some studies show that large-diameter stents are more frequently associated with severe complications such as hemorrhage, perforation, fistula, and fever. On the other hand, dysphagia due to stent migration was observed more frequently in patients with a small-diameter stent [47]. Another study by Thomas confirmed that the smaller diameter of stents (<20 mm) was related to stent migrations in malignant strictures [48].

When discussing strategies for palliative management of malignant dysphagia, the main goal of treatment and the patient’s life expectancy must be considered. A significant proportion of patients with esophageal cancer are diagnosed at an advanced stage of the disease. Therefore, palliative treatment may be the only recommended solution Since life expectancy in this population is very low, the main goal of treatment is to improve quality of life. Studies show that dysphagia is one of the most important factors contributing to the deterioration of quality of life [49]. Madhusudhan and colleagues assessed improvement in dysphagia relief and Qol after ES. The European Organization for Research and Treatment of Cancer (EORTC) QLQ-C30 (version 3) and EORTC QLQ -Esophagus (OES) 18 questionnaires were used. Improvement in quality of life and relief of dysphagia were observed until the last follow-up (8 weeks after the procedure). The median survival was 4 months [50].

ES was shown to provide rapid relief of dysphagia, but the effects may be short-term and not superior to those observed in patients undergoing brachytherapy or laser treatment [51]. Brachytherapy combined with external beam radiation improved dysphagia scores. Weight gain was also observed; however, 39% of the patients required nasogastric tube feeding and 16% required hospitalization for supportive care [52]. A comparable population was studied by researchers from Roterdam. General and disease-specific questionnaires were used to compare patients treated with a single dose of brachytherapy compared to those with ES. Brachytherapy was associated with better quality of life. Quality of life deteriorated over time in both groups, but the deterioration was more pronounced in the ES group [53]. A recent systematic review has shown that ES in combination with radiotherapy compared with ES alone may improve survival without additional risk of complications [54]. Although dysphagia relief in stented patients is observed and oral feeding may be restored, the influence on nutritional status is controversial. A meta-analysis by Nagaraja showed that ES led to the relief of dysphagia, weight gain, and higher albumin levels at follow-up in cancer patients [46]. On the other hand, further data showed that ES resulted in survival of 2.4 to 143.5 months in patients during neoadjuvant therapy and had no effect on improving weight, BMI, or albumin levels [44]. In a retrospective study Song et al. showed that endoscopic gastrostomy placement resulted in better nutritional status than esophageal stenting; however, with no differences in overall survival. Decreased body weight and albumin levels were observed in both groups at follow-up [55]. Dubecz et al. showed a survival of 104 days after ES for esophageal cancer and 48 days for lung cancer. A total of 20% of the patients survived less than 1 month [56]. In the study of Lecleire, the median survival with ES was 76 days. Low BMI and albumin levels predicted mortality after the procedure [57]. On the other hand, Gray and colleagues showed a survival of 84 days and no effect of BMI, caloric intake, and swallowing capacity on survival [58]. This was confirmed in our study. The median survival with malignant diagnosis was short (90 days). Nutritional status had no effect on complication rates or survival.

### Limitations of the Study

Due to the nature of a retrospective study, some limitations must be mentioned. The impact of the nutritional status on complications and survival was analyzed; however, complete data on nutrition interventions prior of after the procedure were not available. Data on cancer treatment before ES were also not included in the analysis. The survival time of cancer patients was short; therefore, the effects of ES on nutritional status in the follow-up were not analyzed. Long-term observations of patients with benign diagnoses should be planned in future studies.

## 5. Conclusions

Endoscopic stenting of the esophagus is a relatively safe procedure with low complication rates that can be managed endoscopically. Significant improvement in dysphagia symptoms can be observed after the procedure. Although the prevalence of malnutrition is high in patients eligible for stenting, nutritional status does not affect complication rates and survival.

## Figures and Tables

**Figure 2:**
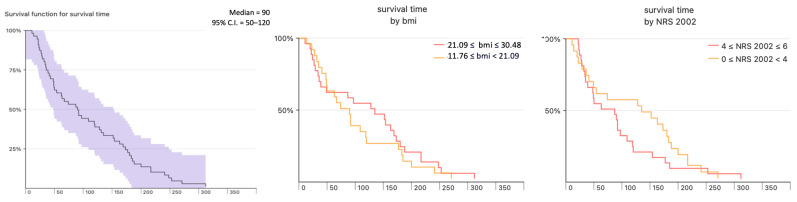
Survival curve of patients with malignant stenosis (overall, subdivided in relation to the median cut-off values of BMI and NRS 2002).

**Table 1:** Patients’ characteristics and indications for ES.

	Benign *n* = 25	Malignant *n* = 56
Age (median)	61.0 (IQR- 47)	67.5 (IQR- 16)
Indications %	Post-inflammatory stenosis 32%	Esophageal cancer 75% Gastric cancer 5.4% Extrinsic compression 20%
	Stenosis after surgery 8.0%	
	Chemical burn 24%	
	Perforation 36%	
Dysphagia	Grade 0 8.0%	Grade 0 1.8%
	Grade 1 8.0%	Grade 1 1.8%
	Grade 2 36%	Grade 2 20%
	Grade 3 16%	Grade 3 37%
	Grade 4 16%	Grade 4 27%
	Undefined 16%	Undefined 12%

**Table 2:** Influence of patients’, stent, and tumor characteristics on complications.

	Complications Overall	Tissue Overgrowth	Migration	Bleeding	Fistula Formation
Age (*p*-value)	0.767	0.584	0.525	0.584	0.513
66 ≤ Age ≤ 89 *n* (%)	12 (28.6%)	2 (4.8%)	5 (11.9%)	2 (4.8%)	1 (2.4%)
21 ≤ Age < 66 *n* (%)	10 (25.6%)	3 (7.7%)	3 (7.7%)	3 (7.7%)	2 (5.1%)
Sex (*p*-value)	0.669	0.631	0.705	0.145	0.925
Female *n* (%)	6 (24.0%)	1 (4.0%)	2 (8.0%)	3 (12.0%)	1 (4.0%)
Male *n* (%)	16 (28.6%)	4 (7.1%)	6 (10.7%)	2 (3.6%)	2 (3.6%)
Length of stent (*p*-value)	0.936	0.175	0.75	0.712	0.975
10 ≤ length ≤ 16 *n* (%)	15 (27.8%)	2 (3.7%)	5 (9.3%)	3 (5.6%)	2 (3.7%)
6 ≤ length < 10 *n* (%)	7 (26.9%)	3 (11.5%)	3 (11.5%)	2 (7.7%)	1 (3.8%)
Diameter (*p*-value)	0.157	0.687	0.04	0.687	0.07
2.2 ≤ diameter ≤ 2.4 *n* (%)	8 (20.0%)	3 (7.5%)	1 (2.5%)	3 (7.5%)	0 (0.0%)
1.8 ≤ diameter < 2.2 *n* (%)	13 (34.2%)	2 (5.3%)	6 (15.8%)	2 (5.3%)	3 (7.9%)
Localization of stenosis ** (*p*-value)	0.409	0.174	0.828	0.872	0.058
Lower *n* (%)	8 (33.3%)	2 (8.3%)	3 (12.5%)	1 (4.2%)	0 (0.0%)
Middle *n* (%)	5 (18.5%)	1 (3.7%)	2 (7.4%)	2 (7.4%)	0 (0.0%)
Upper *n* (%)	9 (32.1%)	2 (7.1%)	3 (10.7%)	2 (7.1%)	3 (10.7%)
Indication (*p*-value)	0.131	0.304	0.669	0.587	0.238
Benign *n* (%)	4 (16.0%)	0 (0.0%)	3 (12.0%)	1 (4.0%)	0 (0.0%)
Cancer *n* (%)	18 (32.1%)	5 (8.9%)	5 (8.9%)	4 (7.1%)	3 (5.4%)
Histopathology (*p*-value)	0.063	0.073	0.404	0.521	0.546
AC *n* (%)	7 (53.8%)	2 (15.4%)	2 (15.4%)	2 (15.4%)	0 (0.0%)
SCC *n* (%)	7 (30.4%)	3 (13.0%)	2 (8.7%)	1 (4.3%)	1 (4.3%)
Extra * *n* (%)	1 (9.1%)	0 (0.0%)	0 (0.0%)	1 (9.1%)	1 (9.1%)
Missing data *n* (%)	7 (20.6%)	0 (0.0%)	4 (11.8%)	1 (2.9%)	1 (2.9%)

Diameter and length of the stenosis are provided in centimeters. * Extra—external compression of esophagus caused by non-esophageal cancer. ** Proximal stenosis onset estimated during endoscopic procedure.

**Table 3:** Patient’s nutritional status and complications of stenting.

	Complications (Overall)	Tissue Overgrowth	Migration	Bleeding	Fistula Formation
BMI (*p*-value)	0.185	0.304	0.089	0.165	0.556
21,1 ≤ bmi ≤ 30,5 *n* (%)	12 (31.6%)	1 (2.6%)	5 (13.2%)	4 (10.5%)	2 (5.3%)
11,8 ≤ bmi < 21,1 *n* (%)	7 (18.4%)	3 (7.9%)	1 (2.6%)	1 (2.6%)	1 (2.6%)
Dysphagia start (*p*-value)	0.091	0.453	0.687	0.651	0.19
<3 *n* (%)	4 (14.8%)	1 (3.7%)	2 (7.4%)	1 (3.7%)	0 (0.0%)
≥3 *n* (%)	16 (32.7%)	4 (8.2%)	5 (10.2%)	3 (6.1%)	3 (6.1%)
Missing *n* (%)	2 (40.0%)	0 (0.0%)	1 (20.0%)	1 (20.0%)	0 (0.0%)
Dysphagia relief (*p*-value)	0.913	0.549	0.667	0.083	0.922
<3 *n* (%)	13 (27.1%)	2 (4.2%)	5 (10.4%)	5 (10.4%)	2 (4.2%)
≥3 *n* (%)	7 (25.9%)	2 (7.4%)	2 (7.4%)	0 (0.0%)	1 (3.7%)
Missing *n* (%)	2 (33.3%)	1 (16.7%)	1 (16.7%)	0 (0.0%)	0 (0.0%)
NRS 2002 (*p*-value)	0.604	0.925	0.186	0.714	0.282
<3 *n* (%)	6 (30.0%)	1 (5.0%)	3 (15.0%)	1 (5.0%)	0 (0.0%)
≥3 *n* (%)	13 (24.1%)	3 (5.6%)	3 (5.6%)	4 (7.4%)	3 (5.6%)
Missing	3 (42.9%)	1 (14.3%)	2 (28.6%)	0 (0.0%)	0 (0.0%)
GLIM (*p*-value)	0.486	0.734	0.086	0.304	0.499
1 *n* (%)	1 (12.5%)	0 (0.0%)	1 (12.5%)	0 (0.0%)	0 (0.0%)
2 *n* (%)	15 (26.8%)	4 (7.1%)	3 (5.4%)	5 (8.9%)	3 (5.4%)
Incomplete data	6 (35.3%)	1 (5.9%)	4 (23.5%)	0 (0.0%)	0 (0.0%)

Median survival of patients after ES with malignant diagnosis was 90 days (95% C.I. = 50–120); 20% of patients survived less than 30 days (Figure 2). Nutritional status: BMI (*p* = 0.484), NRS 2002 score (*p* = 0.963), and GLIM (*p* = 0.872), as well as stage of dysphagia at the time of stenting, did not affect overall survival. No statistical differences were observed in survival between patients subdivided in relation to the median cut-off values of BMI (*p* = 0.396) and NRS 2002 (*p* = 0.273). (BMI—body mass index, NRS 2002—Nutrition Risk Screening, GLIM—Global Leadership Initiative on Malnutrition).

## Data Availability

Not applicable.
